# Preparation of ^68^Ga-PSMA-11 with a Synthesis Module for Micro PET-CT Imaging of PSMA Expression during Prostate Cancer Progression

**DOI:** 10.1155/2018/8046541

**Published:** 2018-04-26

**Authors:** Yuebing Wang, Guoqiang Shao, Jianping Wu, Can Cui, Shimin Zang, Fan Qiu, Ruipeng Jia, Zizheng Wang, Feng Wang

**Affiliations:** ^1^Department of Biochemistry, School of Medicine, Nankai University, Tianjin 300071, China; ^2^Department of Nuclear Medicine, Nanjing First Hospital, Nanjing Medical University, Nanjing 210006, China; ^3^Department of Urology Surgery, Nanjing First Hospital, Nanjing Medical University, Nanjing 210006, China

## Abstract

**Objective:**

To synthesize ^68^Ga-Glu-urea-Lys(Ahx)-HBED-CC (^68^Ga-PSMA-11) with a synthesis module and investigate PET-CT imaging to monitor PSMA expression during prostate cancer (PCa) progression and tumor growth in mice bearing subcutaneous PCa xenografts.

**Method:**

The radiochemical purity and stability of  ^68^Ga-PSMA-11 were determined via radio-HPLC. The PCa cell lines of different PSMA expression levels (PC3, VCAP±, CWR22RV1+, and LNCaP++) were selected to mimic the PCa progression. ^68^Ga-PSMA-11 biodistribution was studied by dissection method and* in vivo* imaging with micro PET-CT. The expression levels of PSMA in tumor cells and tissues were analyzed by immunofluorescence, flow cytometry, and western blot. The correlation between PSMA expression and radio-uptake was also evaluated. 2-PMPA preadministration served as a block group.

**Results:**

The radiochemical purity of  ^68^Ga-PSMA-11 was 99.6 ± 0.1% and stable* in vitro* for 2 h. The equilibrium binding constant (Kd) of  ^68^Ga-PSMA-11 to LNCaP, CWR22Rv1, PC-3, and VCAP cells was 4.3 ± 0.8 nM, 16.4 ± 1.3 nM, 225.3 ± 20.8 nM, and 125.6 ± 13.1 nM, respectively. Results of tumor uptake (% ID and % ID/g or % ID/cm^3^) of  ^68^Ga-PSMA-11 in biodistribution and micro PET imaging were LNCaP > CWR22RV1 > PC-3 and VCAP due to different PSMA expression levels. It was confirmed by flow cytometry, western blot, and immunofluorescence. Tumor uptake (% ID/cm^3^) of  ^68^Ga-PSMA-11 increased with the tumor anatomical volume in quadratic polynomial fashion and reached the peak (when tumor volume was 0.5 cm^3^) earlier than tumor uptake (% ID). Tumor uptake (% ID/cm^3^) of  ^68^Ga-PSMA-11 based on functional volume correlated well with the PSMA expression in a linear manner (*y* = 9.35*x* + 2.59, *R*^2^ = 0.8924, and *p* < 0.0001); however, low dose 2-PMPA causes rapid renal clearance of increased tumor/kidney uptake of  ^68^Ga-PSMA-11.

**Conclusions:**

The ^68^Ga-PSMA-11 PET-CT imaging could invasively evaluate PSMA expression during PCa progression and tumor growth with % ID/cm^3^ (based on functional volume) as an important index. Low dose 2-PMPA preadministration might be a choice to decrease kidney uptake of  ^68^Ga-PSMA-11.

## 1. Introduction

In spite of great efforts and recent advances in early diagnosis and surgical intervention, prostate cancer (PCa) remains the most commonly diagnosed cancer and second leading cause of cancer-related death in men over 40 years [1]. Therapeutic effects for localized PCa in patients of stages I, II, and III are relatively fine by standard employed treatments. PCa patients with relapse, distant metastasis (stage IV), and high risk of PCa progression and/or death are all considered as advanced PCa [2, 3]. Most of them become resistant to hormonal approach and developed a metastatic castration-resistant prostate cancer (mCRPC) shortly after androgen deprivation therapy. Docetaxel therapy, most widely used for mCRPC, is of decreasing therapeutic efficacy due to lack of specificity and associated side effects. It becomes a main challenge to effectively diagnose and select appropriate treatment options for advanced PCa and mCRPC.

Prostate-specific membrane antigen (PSMA), a type II extracellular glycoprotein, is highly expressed in most prostate cancer cells and low or negative in most normal organ systems [4–6]. As a consequence, PSMA represents an ideal target for specific prostate cancer imaging and endoradiotherapy; moreover, PSMA-targeting antibodies, inhibitors, and peptides have been rapidly developed for prostate cancer targeting endoradiotherapy and chemotherapy. Importantly, PSMA is presented on the cell surface or in the cell plasma, and it is not shed into the circulation [7], so it is not a convenient biomarker to be examined in blood as serum PSA. Pathology diagnosis served as the gold standard and decisions for PSMA-targeting therapies are mainly made on the basis of PSMA expression in primary tumors. However, PSMA expression levels vary in different prostate cancers. The PSMA expression is highly upregulated in advanced, poorly differentiated PCas and increases with tumor aggressiveness [6, 8]. The expression levels of PSMA are reported to be associated with tumor grade and clinical outcome [9, 10]. Biopsies of recurrent or metastasis lesions are infrequently performed. Pretreatment imaging or biomarkers for patient selection and response predicting to PSMA-directed therapy are urgently needed.

Glu-urea-Lys(Ahx)-HBED-CC(Glu-urea-lys-(*N,N*′-bis-[2-hydroxy-5-(carboxyethyl)benzyl]ethylenediamine-*N,N*′-diacetic acid)) containing a HBED-CC chelator is used for the preparation of  ^68^Ga-PSMA-11. ^68^Ga-PSMA-11 prepared in standard protocol at 95°C can bind to PSMA with high affinity and specificity [11–13]. In this study, we prepared ^68^Ga-PSMA-11 with semiautomatic module. PCa cell lines with different PSMA expression levels were selected to mimic PCa progression. ^68^Ga-PSMA-11 imaging was performed to monitor PSMA expression changes during tumor growth. The aim of this study was to evaluate the feasibility of  ^68^Ga-PSMA-11 PET-CT imaging to reflect tumor progression and growth for further PSMA-targeted therapy in the clinic.

## 2. Materials and Methods

### 2.1. Materials

LNCaP, CWR22Rv1, PC-3, and VCAP prostate cancer cell lines were obtained from the American Type Culture Collection (ATCC, Manassas, VA). Nude mice (male, 14–16 g, 5-6 wk) were supplied by the Chinese Academy of Medical Sciences, Shanghai, China. Primary antibody is anti-PSMA antibody (YPSMA-1, Abcam, USA). Secondary antibody is goat anti-rabbit IgG-HRP (Santa Cruz biotechnology, USA). DAPI (4′,6-Diamidino-2-Phenylindole, Dihydrochloride) was purchased from Life Technologies (USA). All other chemicals and cell-culture reagents were purchased from Sigma-Aldrich or Gibco Corporation. The radio-high performance liquid chromatography (radio-HPLC) was performed using a LabAlliance system (Scientific Systems, Inc., State College, PA) equipped with a *β*-ram IN-US detector and Zorbax C18 column (4.6 mm × 250 mm, 300 A pore size). ^68^Ge-^68^Ga generator and automated module for ^68^Ga labeling were offered by ITG Corporation (Germany). We also used Micro PET/CT (Inveon, Siemens, Germany); *γ*-counter (TY6017, Wizard, PerkinElmer, USA); flow cytometry (Cytomics FC 500, Beckman, USA); Olympic BX51 fluorescence microscope (Olympus America Inc., Center Valley, PA).

### 2.2. Cell Culture and Animal Model Establishment

The LNCaP and CWR22Rv1 cells were cultured in RPMI medium (ATCC, Manassas, VA). PC-3 and VCAP cells were grown in the F-12K medium (ATCC, Manassas, VA). All medium was supplemented with 10% fetal bovine serum (FBS, ATCC) and 1% antibiotic solution (Sigma-Aldrich, St. Louis, MO). Cells were cultured at 37°C in a humidified atmosphere of 5% CO_2_ in air. Cells were grown as monolayers and were harvested or split when they reached 80% confluence to maintain exponential growth. One day before the cell binding experiment, PCa cells were seeded in 24-well plates.

All animal experiments were conducted in accordance with standards of the Institutional Animal Care and Utilization Committee of Nanjing Medical University. 5 × 10^6^ PC-3, LNCaP, CWR22Rv1, or PCa cells in 50% Matrigel (Becton Dickinson, Heidelberg, Germany) were injected subcutaneously into the mice left armpit to establish animal models.

### 2.3. Synthesis of ^68^Ga-PSMA-11 with the Synthesis Module and* In Vitro* Stability Study

^68^Ga-PSMA-11 was semiautomatically synthesized using the labeling module (iTG) as shown in Supplemental Figure 1. The system was lead shielded and the synthesis was performed in Grade IV lab. In brief, the C18 cartridge was preconditioned by passing first 5 ml 70% ethanol through followed by 10 ml PBS via “A” indicated on Supplemental Figure 1. 5 *μ*g (5.3 nmol) of HBED-CC-Lys-CO-Glu in 1 ml of sodium acetate buffer (0.25 M) was injected manually into the reaction vial. ^68^GaCl_3_, eluted from ^68^Ge-^68^Ga generator by slow injection of 4 ml of 0.05 M HCl via “C,” was sent to the reactor and kept at 95°C for 5 min. The reaction mixture was pushed through the C18 cartridge and the effluent was collected in the waste vial. The product that remained on the C18 cartridge will pass through a 0.22 *μ*m pore size filter by ethanol (60%) injection via “B” and will be collected in the product vial. Ethanol concentration in product was no more than 10% before use [14].

Radiochemical yield of  ^68^Ga-PSMA-11 prepared using synthesis module is calculated by comparing the activity of  ^68^Ga-PSMA-11 with the total activity. The* in vitro* stability of  ^68^Ga-PSMA-11 was studied by incubating in NaOAC/HAC buffer solutions with PH value of 4, 5.5, and 7.4. Radiochemical stability of  ^68^Ga-PSMA-11 was analyzed using radio-HPLC 2 h after incubation at 37°C. Radio-HPLC was conducted on an LC-20AT system (Shimadzu, Japan) equipped with Zorbax-Rx C18 HPLC column (4.6 × 250 mm). The mobile phase was isocratic with solvent A (water containing 0.05% trifluoroacetic acid, TFA) and solvent B (acetonitrile with 0.05% TFA). Gradient mobile phase was from 0% B and 100% A at 0 min to 100% B and 0% A at 30 min. The flow rate was 1 mL/min.

### 2.4. Scatchard Analysis of ^68^Ga-PSMA-11

Scatchard analysis was the standard method for analyzing the equilibrium binding parameters and the dissociation constant (Kd) of a radiolabeled drug with its receptor. The experiment was performed as reported [15]. Briefly, LNCaP, CWR22Rv1, PC-3, and VCAP cells were cultured in 24-well plates for 4 h. They were then coincubated with gradient concentrations of  ^68^Ga-PSMA-11 (0.02–40 nm) in 1 ml RPMI1640 containing 0.5% w/v bovine serum albumin for another 3 h on ice. Cells were washed three times with ice-cold PBS and the cell-associated radioactivity was calculated using *γ*-counter. Nonspecific binding was determined by coincubation with overdose (100 *μ*M) cold PSMA-11. The specific binding ^68^Ga-PSMA-11 was plotted against the bound radioligand/free radioligand ratio. Data were analyzed by linear regression to determine PSMA antigen density per cell and the slope of the line is equal to −1/Kd.

### 2.5. Biodistribution Studies

^68^Ga-PSMA-11 biodistribution studies were performed when the long diameter of tumors reached 0.7–0.8 cm. Mice bearing subcutaneous PCa (LNCaP, CWR22Rv1, and PC-3) xenografts were anesthetized by isoflurane inhalation and injected 1.0 MBq of  ^68^Ga-PSMA-11 in 100 *μ*L of saline via tail vein. Mice bearing LNCaP xenografts with 10 min preinjection of 250 *μ*g (high dose) and 2.5 *μ*g (low dose) 2-(phosphonomethyl) pentane-1,5-dioic acid (2-PMPA) served as the PSMA-block group. Mice were sacrificed for biodistribution analysis 1 h (*n* = 5 for each) after tail vein injection of  ^68^Ga-PSMA-11. Important organs and tumor xenografts were harvested, weighed, and counted on *γ*-counter (PerkinElmer Wizard 1480, Shelton, CT). The percentage of injected dose per organ (% ID) and percentage of injected dose per gram of tissue (% ID/g) were calculated.

### 2.6. ^68^Ga-PSMA-11 Micro PET-CT Imaging in Mice Bearing PCa Xenografts of Different Cell Line and Tumor Size

When the long diameter of LNCaP, CWR22Rv1, and PC-3 tumor xenografts reached 0.7–0.8 cm, mice bearing PCa xenografts (*n* = 4) were imaged using micro PET-CT (Inveon, Siemens) 1 h after tail vein injection of  ^68^Ga-PSMA-11 (5.5 MBq in 100 *μ*l of saline). The energy window ranged from 384 keV to 638 keV. PET scans were acquired for 10 min in list mode followed by CT scans for 5 min. PET images were reconstructed with three-dimensional ordered subsets expectation maximum (3D-OSEM/MAP) algorithm and CT images were reconstructed with Filtered backprojection (FBP). Micro PET-CT images were analyzed using the Inveon Research Workplace software. Region of interest (ROI) and volume of interest (VOI) were drawn slice by slice based on micro PET and micro CT images, respectively, according to our previous study and another report [16, 17]. Briefly, tumor boundary was drawn on micro PET images in transversal section slice by slice with the threshold of 40% of the maximum of standard uptake value (SUV max). Tumor uptake (% ID) was obtained from uptake values within VOI while tumor uptake (% ID/cm^3^) on micro CT or micro PET was calculated by dividing tumor uptake (% ID) with tumor volume obtained from micro CT (anatomical volume, Vct) or micro PET (functional volume, Vpet).

For the blocking study, mice (*n* = 4) were preinjected with 250 *μ*g or 2.5 *μ*g of 2-PMPA in 100 *μ*L saline 10 min prior to the injection of  ^68^Ga-PSMA-11. To monitor changes of tumor uptake of  ^68^Ga-PSMA-11 and PSMA expression during tumor growth, nude mice bearing different size of LNCaP xenografts (*n* = 9, from 0.07 cm^3^ to 1.72 cm^3^) accepted ^68^Ga-PSMA-11 micro PET-CT imaging.

### 2.7. Flow Cytometry, Western Blot, and Immunofluorescence Assessment for PSMA Expression in Tumor Cells and Tumor Tissues

To analyze PSMA expression, cultured PCa cells or cell suspensions from tumor tissues (3 × 10^5^) were incubated with the primary antibody (1 : 50) at 4°C for 2 h. After washing with PBS (1x) three times, cells were incubated with secondary antibody at 4°C for 30 min. After washing and resuspension with PBS, the cells were analyzed using flow cytometry.

Western blot analysis of PSMA cells were performed as reported [18]. In brief, total protein lysates were prepared and quantified using the Bio-Rad DC Protein Assay. The samples were prepared for gel electrophoresis and each well of the gel was loaded with 15 *μ*g protein to run for 40 min. The blotting step was carried out with the Bio-Rad TransBlot®Turbo™ RTA Transfer kit. The primary antibody against PSMA (Dako M3620, monoclonal mouse) and HRP-labeled anti-mouse secondary antibodies (Dako, dilution 1/1000) were used. Chemiluminescent substrate (ECL system by Amersham Bioscience, Freiburg, Germany) was used to determine relative density.

Frozen tumor tissue slices (LNCaP, CWR22Rv1, thickness of 5 *μ*m) were fixed with 4% paraformaldehyde for 1 hour and then were blocked with 5% bovine serum albumin for 45 min. After being incubated with the primary antibody overnight at 4°C and three washes with PBS, cells were incubated with the AlexaFluor488 labeled-secondary antibody (Invitrogen) for 45 min and visualized with a fluorescence microscope.

### 2.8. Statistical Analysis

All data were expressed as the means plus standard deviation.* Student*'*s t-test* was used for statistical analysis. Statistical analysis was performed by one-way analysis of variance (ANOVA) followed by the Newman-Keuls test for multiple comparisons. The level of significance was set at *p* < 0.05. For linear and quadratic polynomial regression analysis, we used the GraphPad Prism 5 software (GraphPad Software Inc., La Jolla, CA).

## 3. Results

### 3.1. *In Vitro* Characteristics of ^68^Ga-PSMA-11

**Figure 1:**
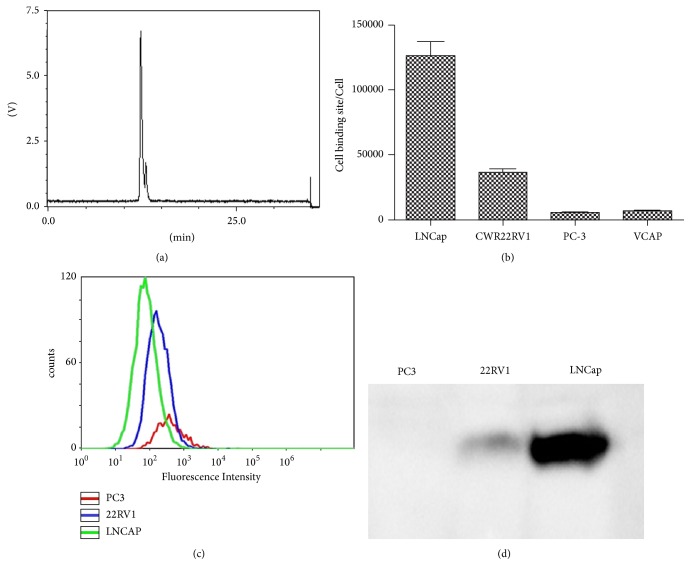
(a) The radio-HPLC results of  ^68^Ga-PSMA-11 prepared using the synthesis module 2 h after its preparation. (b) The cell binding sites of  ^68^Ga-PSMA-11 in LNCaP, CWR22Rv1, PC-3, and VCAP cells from the saturation binding experiments. (c) Representative flow cytometry image of PSMA expression levels. (d) Representative western blot image of PSMA expression levels.

Radioactivity in the waste vial and cartridge in all the reactions was less than 5%. The radiochemical yield of  ^68^Ga-PSMA-11 from the synthesis module was more than 95%.  Figure 1(a) displays the radio-HPLC result of the ^68^Ga-PSMA-11 immediately after preparation at 95°C. There were two peaks with averaged retention time of 11.1 min and 11.5 min (peak 1 and peak 2), which was corresponding to different diastereomers of  ^68^Ga-PSMA-11. The peak 2 fraction of  ^68^Ga-PSMA-11 (7.1%  ± 2.4%) is significantly less than peak 1 (*p* < 0.001). The radiochemical purity of  ^68^Ga-PSMA-11 (including all diastereomers) was as high as (99.6 ± 0.1)% and stable* in vitro* for 2 h (PH 7.4). The peak 1 fraction or radiochemical purity in buffer solution (PH 7.4) at 2 h* in vitro* was insignificantly different from that in buffer solution (PH 4.0 or PH 5.5).

### 3.2. ^68^Ga-PSMA-11 Binding to PSMA-Expressing PCa Cells

The saturation binding experiments of the binding of  ^68^Ga-PSMA-11 to LNCaP, CWR22Rv1, PC-3, and VCAP cells* in vitro* showed that the equilibrium dissociation constant (Kd) of  ^68^Ga-PSMA-11 to these cell lines was 4.3 ± 0.8 nM, 16.4 ± 1.3 nM, 225.3 ± 20.8 nM, and 125.6 ± 13.1 nM, respectively. The number of  ^68^Ga-PSMA-11 binding sites for PCa cell lines was shown in Figure 1(b). The results were confirmed by cell-based flow cytometry (Figure 1(c)) and western blot assays (Figure 1(d)). The binding sites of  ^68^Ga-PSMA-11 were highest for LNCaP, modest for CWR22RV1, and lowest or even negative for PC-3 and VCAP. The binding specificity of  ^68^Ga-PSMA-11 to PSMA was confirmed by blocking study with overdose cold PSMA-11.

### 3.3. ^68^Ga-PSMA-11 Biodistribution and PSMA Expression in Cell Suspension from Tumor Tissues

**Figure 2:**
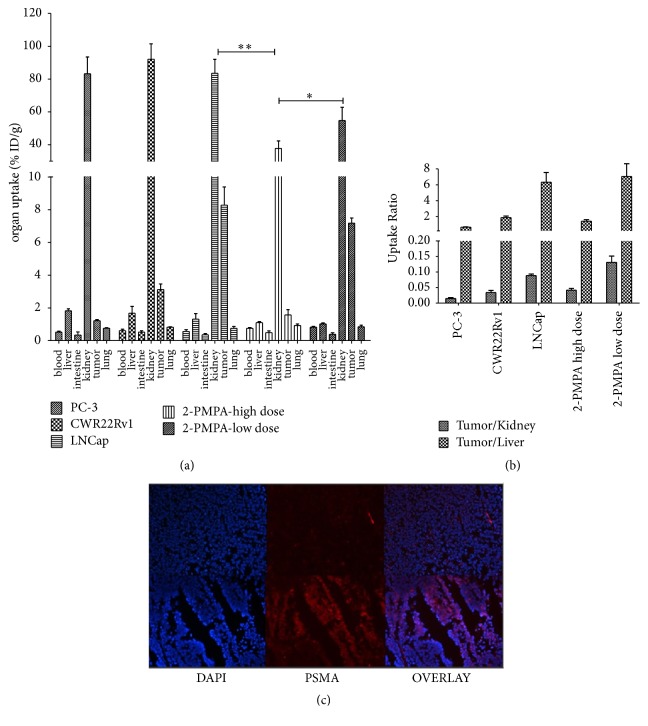
(a) Distribution of  ^68^Ga-PSMA-11 in nude mice bearing PC-3, CWR22Rv1, and LNCaP xenografts 1 h after the intravenous injection. The mice bearing LNCaP xenografts with preinjection of 2.5 *μ*g (low dose) or 250 g (high dose) of 2-PMPA were used as blocking groups (10 min before radiotracer administration). (b) The graph of tumor/kidney and tumor/liver uptake ratio in mice bearing different type of PCa xenografts with or without 2-PMPA. (c) Representative images (original magnification 100x) of CWR22Rv1 and LNCaP cells with anti-PSMA antibody (AlexaFluo488, red) stained and nucleus stained (Hoechst, blue).

Figure 2 showed the biodistribution of  ^68^Ga-PSMA-11 in LNCaP, CWR22Rv1, and PC-3 tumor-bearing mice at 1 h after tail vein injection. LNCaP tumor uptake (% ID/g) of  ^68^Ga-PSMA-11 (7.28 ± 0.82) was significantly higher than that of CWR22Rv1 (3.12 ± 0.35, *p* < 0.01) and PC3 (1.21 ± 0.07, *p* < 0.001) and can be blocked by preinjection of 2-PMPA indicating PSMA binding specificity. The PSMA expression levels in LNCaP and CWR22Rv1 tumor tissues were confirmed by immunofluorescence and it was almost negative in PC-3 cells. Preinjection of 2-PMPA in LNCaP group reduced uptake of  ^68^Ga-PSMA-11 in kidneys (*p* < 0.01). Tumor uptake of  ^68^Ga-PSMA-11 (% ID/g) was reduced significantly by preinjection of high dose of 2-PMPA while it showed insignificant difference after preinjection of low dose 2-PMPA. The tumor/kidney uptake ratio (0.13 ± 0.02) in the 2-PMPA low dose group was significantly higher than that in LNCaP group (0.088 ± 0.005, *p* < 0.001), while the tumor/liver uptake ratio demonstrates insignificant increase after the preinjection of low dose 2-PMPA (from 6.32 ± 1.21 to 7.039 ± 1.37, *p* > 0.05). Preinjection of low dose 2-PMPA in LNCaP group insignificantly reduced tracer distribution of  ^68^Ga-PSMA-11 in salivary glands.

### 3.4. ^68^Ga-PSMA-11 Micro PET-CT Imaging of Tumor with Different PSMA Expression

**Figure 3:**
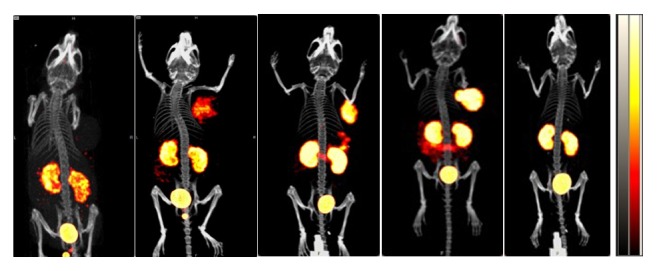
The 3D views of representative micro PET-CT images of nude mice bearing PC-3, CWR22Rv1, LNCaP, and LNCaP xenografts with low dose (2.5 *μ*g) or high dose (250 *μ*g) preinjection of 2-PMPA (from left to right). PET-CT scan was performed 1 h after intravenous injection of  ^68^Ga-PSMA-11 with preinjection of overdose 2-PMPA to verify its specific binding to PSMA.

Figure 3 displays the representative 3D images of mice bearing PC-3, CWR22RV1, and LNCaP xenografts and the block group (10 min preinjection of 250 *μ*g 2-PMPA) 1 h after tail vein injection of  ^68^Ga-PSMA-11. Tumor uptake (% ID/cm^3^) of  ^68^Ga-PSMA-11 in LNCaP (7.63 ± 1.12) was higher than that of CWR22Rv1 (3.54 ± 0.42) and PC-3 (1.21 ± 0.05) based on the micro PET-CT fusion. Preinjection of overdose 2-PMPA blocks tumor uptake of  ^68^Ga-PSMA-11 (*p* < 0.01), whereas slight increase of tumor uptake of  ^68^Ga-PSMA-11 was found after preinjection of low dose 2-PMPA.

### 3.5. ^68^Ga-PSMA-11 Micro PET-CT Imaging during Tumor Growth

**Figure 4:**
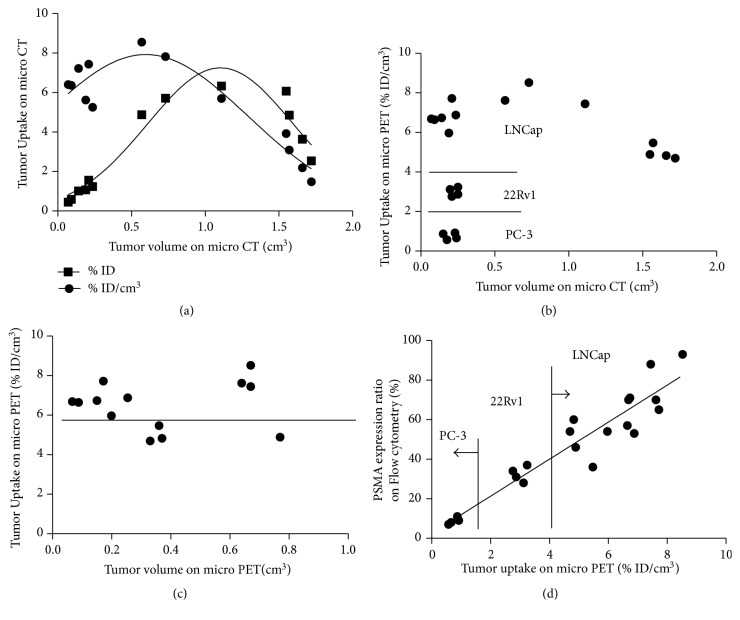
LNCaP tumor uptake (% ID and % ID/cm^3^) of  ^68^Ga-PSMA-11 with tumor growth and relationship with PSMA expression. (a) Graph displays the LNCaP tumor uptake (% ID and % ID/cm^3^) changes with tumor anatomical volume increase. Tumor uptake (% ID/cm^3^) reached peak when anatomical volume was about 0.5 cm^3^, less than that of % ID tumor uptake (1.2 cm^3^). (b) Tumor uptake (% ID/cm^3^) of radiotracer in tumors with different PSMA expression and anatomical volume. When tumor size was less than 0.5 cm^3^, tumor uptake (% ID/cm^3^) of  ^68^Ga-PSMA-11 was LNCaP > CWR22Rv1 > PC-3 and it decreased when tumor grew more than 1.0 cm^3^. (c) LNCaP tumor uptake (% ID/cm^3^) of radiotracer with functional tumor volume changes. LNCaP tumor uptake (% ID/cm^3^) was more than 6.0. In four mice bearing large LNCaP tumor xenografts with anatomical volume > 1.0 cm^3^ and functional volume of about 0.4 cm^3^, the tumor uptake (% ID/cm^3^) is less than 6.0 (below horizontal line). (d) Tumor uptake of  ^68^Ga-PSMA-11 (% ID/cm^3^) based on functional volume correlated well with PSMA expression determined by flow cytometry in a linear manner (*y* = 9.35*x* + 2.59, *R*^2^ = 0.8924, and *p* < 0.0001).

LNCaP tumor uptake (% ID and % ID/cm^3^) of  ^68^Ga-PSMA-11 increased with tumor growth and reached peak when Vct was about 0.5 cm^3^ (% ID/cm^3^) and 1.0 cm^3^ (% ID), respectively (*n* = 13). Vpet was less than Vct due to tumor necrosis and ^68^Ga-PSMA-11 absence (Figure 4(a)). LNCaP tumor uptake (% ID/cm^3^) on micro PET was higher than that of CWR22Rv1 and PC-3 and it started to decrease when Vct was more than 1.0 cm^3^ (Figure 4(b)). Except for slight decrease of tumor uptake of  ^68^Ga-PSMA-11 (% ID/cm^3^) on micro PET in 4 mice with great necrosis and ulcer (below horizontal line in Figure 4(c)), LNCaP tumor uptake of  ^68^Ga-PSMA-11 (% ID/cm^3^) on micro PET was stable. When different cell lines and different tumor size of PCa xenografts were considered, tumor uptake of  ^68^Ga-PSMA-11 (% ID/cm^3^) on micro PET correlated well with PSMA expression determined by flow cytometry in linear manner (*y* = 9.35*x* + 2.59, *R*^2^ = 0.8924, and *p* < 0.0001).

## 4. Discussion

^68^Ga-PSMA-11 is one urea based PSMA-targeted radiotracer with HBED-CC as a chelator. HBED-CC, representing an efficient complexing agent suitable for radiogallium labeling of antibody, peptide at RT, forms three diastereomers (RR, RS, and SS configurations) and conformed to NMR examinations [11, 12, 18–20]. RR is potentially thermodynamically favored and is the main fraction. These diastereomers were stable in solutions of different PH value as shown in our study and demonstrate similar binding specificity and affinity to PSMA [21].

PSMA expression is rarely reported in other tumor cells such as clear cell renal cell carcinoma and transitional cell carcinomas of the bladder and endothelial cells of tumor-associated neovasculature. No endothelial expression of PSMA is observed under physiologic conditions. PSMA expression in PCa varies tremendously in different stage or clinical course. Its evaluation is reported to be associated with clinical management selection, personalized treatment, and clinical outcome [9, 10, 22–24]. Among increasing number of PSMA-targeted agents, ^68^Ga-PSMA-11 can be prepared simply using synthesis module and shows high contrast and favorable targeting specificity. Tumor expression of PSMA was determined by the cell expression density and tumor burden. Cell lines with different PSMA expression level were selected to mimic PCa progression. Cell binding site of  ^68^Ga-PSMA-11 per PCa cell follows LNCaP > CWR22Rv1 > PC-3. Cell-based tracer uptake of  ^68^Ga-PSMA-11was consistent with PSMA expression as revealed by flow cytometry and western blot.

“Liquid biopsy” on circulating tumor cells (CTC) characterization has been studied to address the question of PSMA-targeted therapeutic decision [25]. But PSMA profiling is not only determined by the cell line. Judgement of the PSMA expression level as whole can offer more information for tumor burden evaluation, treatment individualization, and dose determination. ^68^Ga-PSMA-11 micro PET-CT imaging has great potential.

The influence of tumor cell line and tumor size for the evaluation of PSMA expression was seen in Figure 4, and tumor uptake (% ID) of  ^68^Ga-PSMA-11 increased with tumor anatomical volume (less than 1.0 cm^3^) in quadratic polynomial fashion while tumor uptake (% ID/cm^3^) started to decrease when tumor anatomical volume is only 0.5 cm^3^. The reduction or inner absence of radiotracer was mainly due to intratumor irregular tumor natural necrosis, reduced blood perfusion, and interstitial space [26]. Accurate metabolic tumor volume (MTV), reported based on ^18^FDG PET or ^18^F-RGD PET imaging, is promising for target tumor volume delineation of lung cancer and breast cancer [17, 27, 28]. Micro PET is better to provide reproducible results for delineation of tumors characterized by heterogeneous activity distributions while micro CT can reduce influence from intense cardiac uptake and high tracer background. We found that the tumor % ID/cm^3^ changes little during early period and decreased to great degree when necrosis occurs. The possible mechanism is regional ischemia and reduced uptake of  ^68^Ga-PSMA-11. The low radioactivity density (% ID/cm^3^) may suggest the existence of large lesion on CT and necrosis in the same patient. So the tumor burden is little even though the tumor size is large. But this type of lesion may respond less to PSMA-targeted radiotherapy.

As further confirmed by biodistribution studies, ^68^Ga-PSMA-11 PET-CT imaging is excellent candidate for diagnostics of advanced PCa with high PSMA expression. However, elevated uptake and undesired retention of  ^68^Ga-PSMA-11 in the kidneys were expected due to the substantial PSMA expression [29]. It was the major concern for therapeutic application. Besides the tumor, we found that uptake of  ^68^Ga-PSMA-11 in PSMA-expressing kidneys can be significantly blocked by preinjection of 2-PMPA. Increasing tumor/kidney ratio was revealed when the injected dose of 2-PMPA is low. Similar to antifolate pemetrexed which has been used to improve tumor uptake and kidney clearance of radiofolate, 2-PMPA as PSMA inhibitor may have great potential to be used in the PSMA-targeted treatment via coadministration [30].

In summary, the tumor uptake on ^68^Ga-PSMA-11 micro PET (% ID/cm^3^) is correlated well with PSMA expression in a linear manner. ^68^Ga-PSMA-11 micro PET imaging could evaluate the density of PSMA expression, which will be of great value for the selection of PSMA-targeted radiotherapy. As a PSMA inhibitor, 2-PMPA may have the potential to be used to decrease renal uptake and retention of PSMA-targeted reagent.

## Abbreviations

^68^Ga: Gallium-68
PSMA: Prostate-specific membrane antigen
PCa: Prostate cancer
CT: Computed tomography
PET: Positron emission tomography
FDG: Fludeoxyglucose
^18^F: Fluorine-18.

## Supplementary Materials

Supplementary Materials Supplemental Figure*    *1: the synthesis module (Supplemental Figure*    *1(A)) was offered by ITC Company and the synthesis of 68 Ga radiopharmaceuticals was performed in the sterile environment. It is set up lead shielded and consists of the 68 Ge–68 Ga generator, the fluid transfer system (with a 0.22 *μ*m pore size filter to avoid microbial contamination at each transfer valves) (Supplemental Figure*    *1(B)) and reaction, and collection system. Supplement Figure*    *1(C) gives the schematic of the fluidic system in the synthesis module. It consists of a peptide pump, through which the peptide (a) and the generator eluent (c) are passed to reactor vial (heating vial) through V2. The outlet from the reactor is connected to the C18 cartridge via V3 after injection of air via v6. F1, F2, and F3 are three-way valves, introduced to control the direction of liquid and air. So the product and waste can be collected in different vials after the reaction mixture passed through the cartridge.
