# Supplementary material for: Preparation of 68Ga-PSMA-11 with a Synthesis Module for Micro PET-CT Imaging of PSMA Expression during Prostate Cancer Progression
Source: Contrast Media Mol Imaging. 2018 Apr 26;2018:8046541. doi: 10.1155/2018/8046541 (PMC5944242; doi:10.1155/2018/8046541)
Supplement: Supplementary Materials — Supplemental Figure 1: the synthesis module (Supplemental Figure 1(A)) was offered by ITC Company and the synthesis of 68 Ga radiopharmaceuticals was performed in the sterile environment. It is set up lead shielded and consists of the 68 Ge–68 Ga generator, the fluid transfer system (with a 0.22 μm pore size filter to avoid microbial contamination at each transfer valves) (Supplemental Figure 1(B)) and reaction, and collection system. Supplement Figure 1(C) gives the schematic of the fluidic system in the synthesis module. It consists of a peptide pump, through which the peptide (a) and the generator eluent (c) are passed to reactor vial (heating vial) through V2. The outlet from the reactor is connected to the C18 cartridge via V3 after injection of air via v6. F1, F2, and F3 are three-way valves, introduced to control the direction of liquid and air. So the product and waste can be collected in different vials after the reaction mixture passed through the cartridge. [file 8046541.f1.pdf]

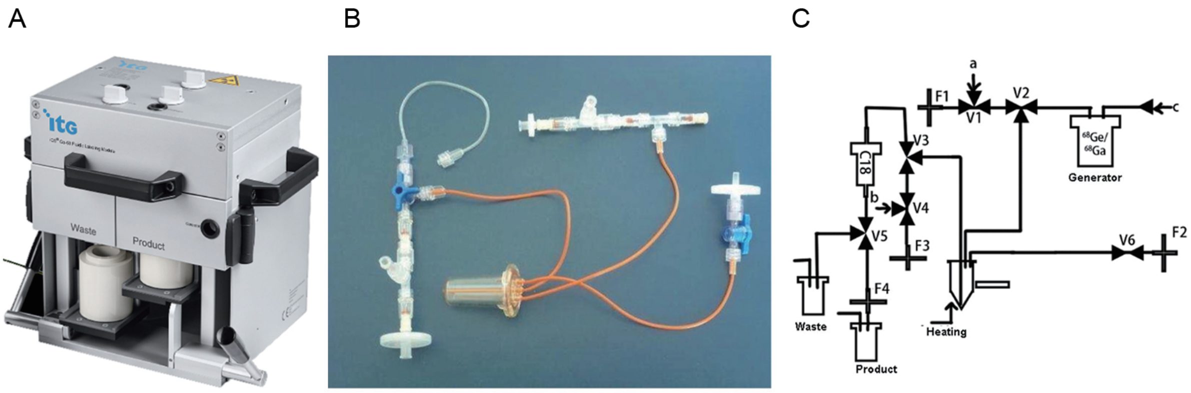

Supplemental Figure 1: The synthesis module (1A) was offered by ITC Company and the synthesis of ^{68}Ga radiopharmaceuticals was performed in the sterile environment. It is set up lead shielded and consists of the ^{68}Ge-^{68}Ga genearator, the fluid transfer system (with a 0.22 µm pore size filter to avoid microbial contamination at each transfer valves) (1B) and reaction, collection system. Supplement Fig 1C gives the schematic of the fluidic system in the synthesis module. It consists of a peptide pump, through which the peptide (a) and the generator eluent (c) are passed to reactor vial (heating vial) through V2. The outlet from the reactor is connected to the C18 cartridge via V3 after injection of air via v6. F1, 2, 3 are three way valves which is introduced to control the direction of liquid and air. So the product and waste can be collected in different vials after the reaction mixture passed through the cartridge.
